# Design Principles for β‑Solenoid Stability via Covalent and Electrostatic Capping Motifs

**DOI:** 10.1021/acs.jpclett.6c01287

**Published:** 2026-05-29

**Authors:** R. J. Eufemio, G. Renzer, J. Lehmann, S. Gerlach, K. Shaw, U. Pöschl, J. Fröhlich-Nowoisky, V. Molinero, M. Bonn, K. Meister

**Affiliations:** † Department of Chemistry and Biochemistry, 1791 Boise State University, Boise, Idaho 83725, United States; ‡ 28308 Max Planck Institute for Polymer Research, 55128 Mainz, Germany; § 28309 Max Planck Institute for Chemistry, 55128 Mainz, Germany; ∥ Department of Chemistry, 7060 The University of Utah, Salt Lake City, Utah 84112-0850, United States

## Abstract

Extended β-solenoid proteins form highly repetitive architectures representing attractive scaffolds for functional biomaterials, yet their termini are intrinsically prone to fraying, and general stabilization strategies remain elusive. Here we identify covalent and electrostatic capping motifs as orthogonal mechanisms that control β-solenoid stability and environmental response. Using fungal and bacterial ice-nucleating proteins as homologous β-solenoid scaffolds, we disentangle how disulfide-mediated and electrostatic capping govern fold integrity under environmental conditions. Disulfide caps in fungi constrain coil unfolding, preserving β-solenoid function under thermal and pH stress but are selectively vulnerable to reductants, which reduce activity by more than 90%. Charged bacterial termini function via electrostatics and are insensitive to reducing agents yet more susceptible to pH and temperature. These results establish terminal capping chemistry as a key handle to tune the stability and environmental robustness of β-solenoid folds, suggesting a promising strategy for the design of repeat-protein scaffolds.

## Graphical abstract

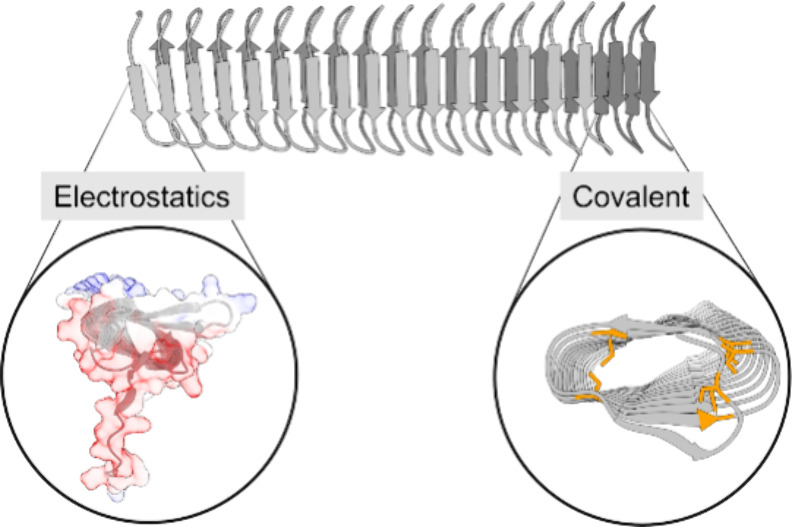

β-Solenoid proteins are widespread in living organisms and account for ∼3% of annotated eukaryotic proteins in UniProt.^1^ They occur across diverse taxa and perform functions like antifreeze activity, molecular recognition, and substrate binding.^2^ These proteins are composed of tandem sequence repeats that assemble into elongated β-sheet folds through the stacking of successive β-strand coils. Their repetitive sequences and extended molecular surfaces have made them attractive scaffolds for engineered protein assemblies such as fibrillar structures, programmable nanomaterials, and protein-based composites.^3,4^ Despite their prevalence and functional diversity, β-solenoid folds present a fundamental stability challenge. Because the fold is built from repeating structural units, the terminal coils contain incomplete β-sheet interactions and are thus prone to fraying or local unfolding.^1,5^ Terminal elements that cap exposed β-strands have been observed in several β-solenoid proteins, but these motifs differ substantially in sequence and structure. Consequently, general principles of β-solenoid terminal stabilization remain poorly defined. Terminal caps identified to date in natural β-solenoids rely primarily on structural irregularities like loops, bulges, and hydrophobic contacts that sterically block intermolecular β-sheet propagation.^1,3^ In de novo β-solenoid design, the absence of effective terminal caps is the dominant reason engineered constructs aggregate or fail to fold.^5,6^ Covalent capping via disulfide bonds has not been explored as a β-solenoid stabilization strategy, despite the well-established role of disulfides in stabilizing β-hairpins and other β-rich folds, including their presence in natural antifreeze proteins^7,8^ and use in engineered peptides to promote β-sheet formation through strand-central disulfides.^9,10^

Ice-nucleating proteins (INPs) provide a direct means to examine how terminal motifs influence β-solenoid stability, as their activity depends on the structural integrity of an extended β-solenoid repeat domain. Predicted AlphaFold3 structures of bacterial INPs from *Pseudomonas syringae* (*Ps*INPs) contain a central repeat domain (CRD) composed of tandem 16-residue repeats that assemble into a continuous β-solenoid fold.^11–13^ Homologous INPs also occur in fungi of the *Mortierellaceae* family.^14^ Although the fungal and bacterial INPs share a conserved CRD architecture, they differ substantially in terminal sequence composition, as shown in Figure 1. Bacterial INPs contain charged residues near the termini of the repeat domain together with a short C-terminal region that was proposed to stabilize terminal coils through electrostatic interactions.^15,16^ In contrast, the fungal INPs lack the charged motifs and instead contain conserved cysteine residues near both ends of the CRD domain of the solenoid. These cysteines suggest an alternative stabilization mechanism in which disulfide bonds constrain the terminal coils of the fold. The AlphaFold3 model (Figure 1) suggests one disulfide bond forms between the two N-terminal cysteines (Cys209 and Cys217). In the C-terminal region, at least four of the six cysteines are positioned such that two stabilizing bonds are likely formed between Cys906 and Cys964, and between Cys968 and Cys972. The two remaining cysteines are not clearly resolved as disulfide-bonded in the model and their interaction cannot be confidently predicted. Here, we go beyond our previous functional characterization of fungal INPs^14^ to identify terminal capping chemistries as a general design principle that controls β-solenoid stability under reducing, alkaline, and thermal stress.

**Figure 1:**
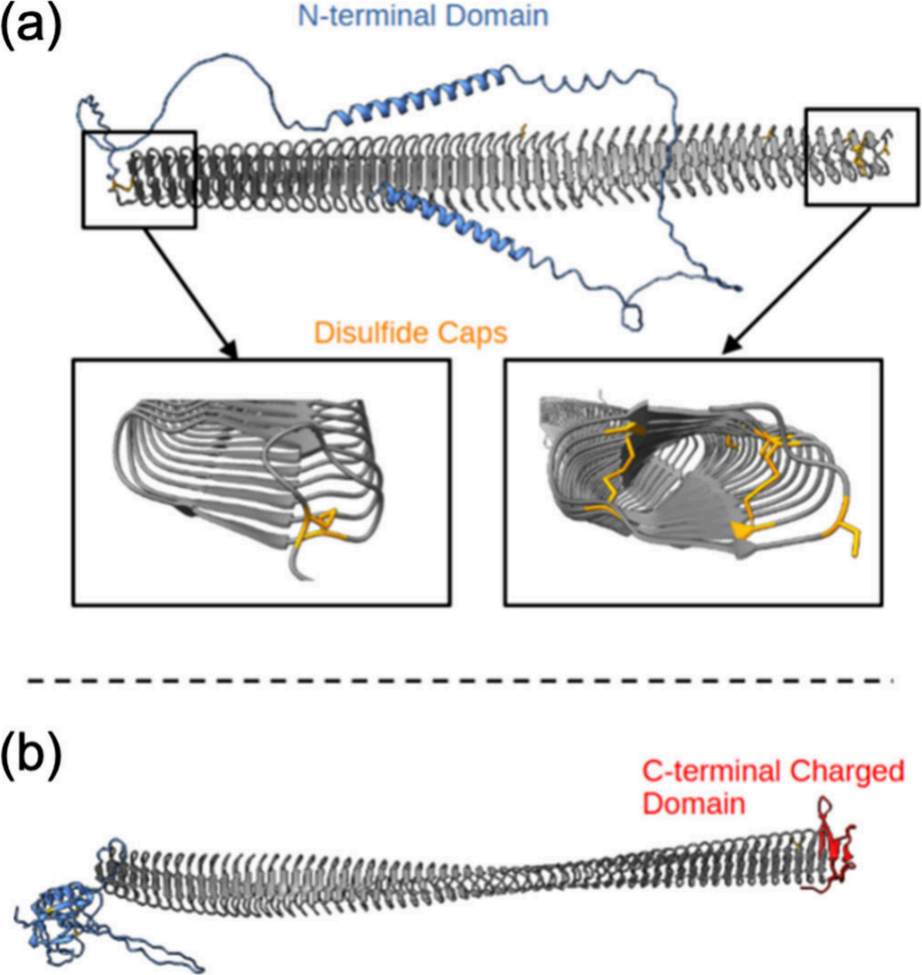
Predicted AlphaFold3 structures of INPs colored according to functional domains. For the fungal INPs from *Entomortierella parvispora* (*En*INP) (a) the N-terminal domain (blue) is followed by the central β-solenoid repeat domain (gray) and the disulfide capping motifs are highlighted in yellow. For bacterial *Ps*INP (b) the N-terminal domain (blue) is followed by the central repeat domain (gray) and the charged C-terminal capping domain (red).

Fungal and bacterial INPs, which share a conserved β-solenoid core but differ primarily in their terminal chemistry of cysteine-rich disulfide capping versus charged C-terminal coils, provide a unique opportunity to isolate how capping mechanism governs stability and to establish disulfide-based capping as a previously unrecognized mode of β-solenoid stabilization. By probing ice nucleation activity under reducing conditions, across alkaline pH, and at elevated temperatures, we determine how these terminal mechanisms govern the integrity of β-solenoid fold required for ice nucleation.

Figure 2 presents cumulative freezing spectra of bacterial ice nucleators (INs) from *P. syringae* and fungal INs from *Entomortierella parvispora* (*En*INPs) measured in water and in the presence of 100 mM dithiothreitol (DTT), a well-known disulfide-reducing agent.^17^ INs from *P. syringae* (0.1 mg/mL) and *E. parvispora* (19 × 10^–5^ mg/mL) were prepared and serially diluted 10-fold. Both preparations yielded saturated active IN populations consistent with previous measurements of maximal ice-nucleation activity.^14,18^ For each concentration, 96 (3 μL) droplets were monitored at a rate of 1 °C/min^19^ until each droplet froze. The cumulative IN number concentration (*N*_m_) was calculated using Vali’s method, and it represents the total number of INs that are active above a given temperature.^20^

**Figure 2:**
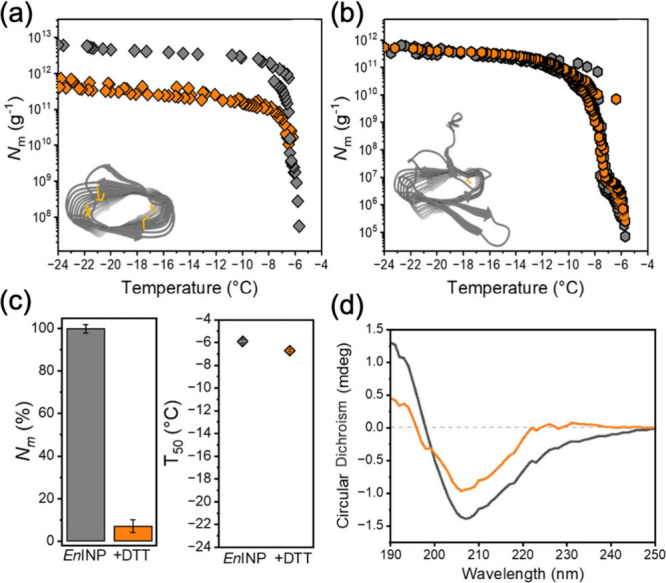
Functional and structural effects of disulfide reduction on INPs. Freezing experiments of aqueous solutions of fungal INs from *E. parvispora* (a) and bacterial INs from *P. syringae* (b) in water (gray) and in the presence of 100 mM DTT (orange). Shown are the cumulative number of INs (*N*_m_) per unit mass of INs vs temperature. The insets show cross sections of the C-terminal region of *En*INP and *Ps*INP. *En*INP has cysteine residues that can form stabilizing disulfide bonds, whereas *Ps*INP has a charged C-terminal capping domain and only a single cysteine residue. (c) Cumulative number of INs and *T*_50_ values at the highest concentration in water and in 100 mM DTT, where *T*_50_ is defined as the temperature at which 50% of droplets freeze. Error bars represent the standard deviation between individual measurements. (d) CD spectra of *En*INPs in water (gray) and in the presence of 20 mM TCEP (orange), indicating structural changes.

For bacterial INs, the cumulative freezing spectra in water and in 100 mM DTT are indistinguishable. Both exhibit a first increase at ∼ −5.7 °C and a second increase at ∼ −7.5 °C followed by a plateau below −9.5 °C, consistent with the presence of two distinct classes of INs with specific activation temperatures as reported previously.^11,18^ The plateaus at temperatures below the increases in *N*_*m*_(T) indicate that there are few INs active at these temperature ranges. The absence of any DTT-induced change shows that reducing conditions do not affect bacterial ice nucleation activity and are consistent with the *Ps*INP sequence, which contains only a single cysteine residue. For fungal INs, the freezing spectra show a single increase in the *N*_m_(*T*) at ∼ −6 °C followed by a plateau below ∼ −8 °C. In the presence of DTT, the freezing spectrum remains similar, but the cumulative number of INs (*N*_*m*_) decreases from ∼ 10^13^ to 10.^12^ At the highest measured concentration, Figure 2c shows similar *T*_50_ values in pure water and 100 mM DTT, ∼ −6 °C and −7 °C, respectively, where T_50_ is the temperature at which 50% of droplets have frozen. However, because freezing of a droplet requires only a single active IN, T_50_ values do not capture changes in the total number of active INs; in this case, a ∼10-fold decrease in *N*_*m*_(T) is accompanied by only a ∼ 1 °C shift in *T*_50_. This illustrates the necessity of analyzing the dilution series, which reveal that the number of fungal INs is reduced by more than 90% in the presence of DTT.

These results demonstrate that reducing conditions largely abolish the ice nucleation activity of fungal INs. *En*INPs contain six cysteine residues near the C-terminal end of the β-solenoid and two cysteines at the beginning of the fold.^14^ These conserved cysteines can form disulfide bonds at both termini, acting as capping structures and stabilizing the fold by preventing terminal uncoiling. Upon addition of DTT, these disulfide bonds are reduced, promoting solenoid uncoiling and resulting in the loss of ice nucleation activity. To support this conclusion, we measured circular dichroism (CD) spectroscopy in water and in the presence of 20 mM tris­(2-carboxyethyl)­phosphine (TCEP), another well-known reducing agent, since DTT has strong absorbance in the far-UV region.^21^ Figure 2d shows CD spectra of *En*INPs in water and in the presence of 20 mM TCEP. The CD spectrum of the untreated sample shows a maximum molar ellipticity at ∼ 190 nm and a minimum at ∼205 nm. Spectral analysis and fold recognition using the web server BeStSel^22^ suggest that *En*INPs contain a high β-sheet content, consistent with their predicted structure. In the presence of TCEP, the CD spectrum of *En*INP shows a reduction of the molar ellipticity at ∼190 nm and an increase in ellipticity at ∼205 nm. These spectral changes suggest alterations in the secondary structure of *En*INPs. Together, these results suggest that reduction of the disulfide bonds destabilized the native fold, leading to conformational changes that cause the observed loss of ice nucleation activity. Overall, our findings establish a direct link between terminal disulfide-mediated capping, β-solenoid stability, and ice nucleation activity under reducing conditions.

Having identified that disulfide reduction impairs the fungal covalent capping mechanisms, and therefore INP function, we next examined how the fungal and bacterial INPs respond to alkaline pH, which was expected to affect electrostatics but not disulfide bonds. To assess how different terminal motifs influence β-solenoid stability and functionality, ice nucleation measurements were performed between pH 6.5 and 12.5 (Figure S1), and the cumulative number of INs, *N*_m_(*T*), was determined from complete dilution series at each condition. Figure 3a shows the cumulative freezing spectra of fungal INs from *E. parvispora* at selected pH values. At pH 6.5 and pH 12, the freezing spectra are similar, with an increase ∼ −6 °C followed by a plateau below ∼ −8 °C. At pH 12.5, the increase near −6 °C persists, while *N*_*m*_(T) decreases by several orders of magnitude, indicating a multiorder-of-magnitude reduction in ice-nucleating activity. Figure 3b shows the freezing spectra of bacterial INs from *P. syringae.* At pH 6.5, two increases in *N*_m_(*T*) are observed at ∼ −3 °C and ∼ −7 °C with plateaus between approximately −4.5 °C and ∼ −7 °C and below ∼ −9.5 °C. At pH 12, the increase ∼ −3 °C is absent, the lower-temperature increase shifts to ∼ −7.5 °C, and *N*_m_(*T*) is reduced. At pH 12.5, *N*_*m*_(T) decreases by more than 2 orders of magnitude, consistent with a near-complete (99.9%) loss of activity. The different responses of fungal and bacterial INs under alkaline conditions are highlighted in Figure 3c, which compares cumulative *N*_m_ values. For bacterial INs, *N*_m_ decreases modestly at pH 11 and then drops sharply at pH 12, with a ∼ 90% reduction, followed by near-complete loss of activity above pH 12.2. In contrast, most fungal INs remain active at pH 11 and at 12, with *N*_*m*_ reduced by only ∼ 30%. At pH 12.2, fungal INs remain partially active, with *N*_*m*_ reduced to ∼ 20%, and are fully inactivated only at higher pH. While the dilution-resolved *N*_m_ values reveal substantial losses in active INs, T_50_ shifts only modestly (Figure S2). Sequence composition of the INPs is consistent with these differences. *Ps*INP contains 58 positively charged and 81 negatively charged residues, with a calculated isoelectric point of ∼ 4.8 (Figures S3 and S4). Charged residues are distributed along one face of the β-solenoid, and within an arginine-rich region near the C-terminus, while the C-terminal cap is enriched in acidic amino acids. *En*INP contains 55 positively charged and 55 negatively charged residues with a calculated isoelectric point of 5.7 (Figures S3 and S4), and lacks both the arginine-rich region and the charged cap. Above pH 10, positively charged residues such as lysine and arginine begin to deprotonate (p*K*_a_ of ∼10.5 and ∼12),^23^ weakening electrostatic interactions that stabilize charged terminal motifs. In bacterial INPs, the loss of positive charge explains the reduced activity observed under alkaline conditions. This interpretation agrees with mutational studies in which removal of terminal charged coils or substitution of basic residues abolishes bacterial ice nucleation activity.^15,16,24^ While these observations support a key role for terminal electrostatics in controlling bacterial IN stability at high pH, we note that in addition to directly affecting terminal electrostatics, pH may also alter membrane-protein interactions that are critical for bacterial INP organization and function.^18,25,26^ Changes in oligomerization state and global charge distribution may further modulate the observed pH response.^18,26,27^ The conserved β-solenoid core and the absence of charged terminal motifs in fungal INPs indicate, however, that differences in terminal chemistry are a primary determinant of their contrasting alkaline stability.

**Figure 3:**
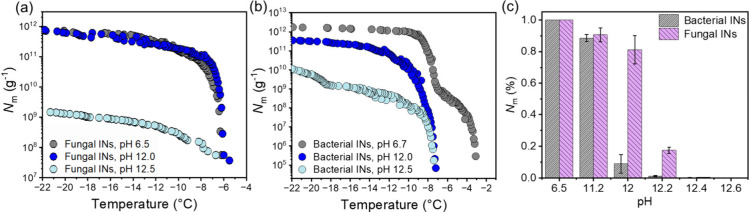
Alkalinity effects on the functionality of INPs. Freezing experiments of aqueous buffered solutions of (a) fungal INs from *E. parvispora* and (b) bacterial INs from *P. syringae* at pH ∼6.5, 12, and 12.5. Shown are the cumulative number of INs (*N*_m_) per unit mass of INs versus temperature. (c) Cumulative number of bacterial and fungal INs as a function of pH. Error bars represent the standard deviation between individual measurements.

To further assess β-solenoid stability, we examined the effect of temperature on bacterial and fungal IN activity. Figure 4 shows cumulative freezing spectra of bacterial and fungal INs that were subjected to temperatures ranging from 20 to 90 °C prior to ice nucleation measurements. As shown in Figure 4a, bacterial INs at 20 °C display two distinct increases in *N*_m_ at ∼ −3 °C and ∼ −7 °C. Heating eliminates the increase near −3 °C and progressively reduces *N*_m_. A measurable reduction is already apparent after heating to 40 °C and becomes more pronounced at higher temperatures. At 60 °C, *N*_m_ is reduced by ∼90% of its initial value, and at temperatures ≥ 70 °C, *N*_m_ decreases further, corresponding to near-complete inactivation. Figure 4b shows that fungal INs at 20 °C exhibit an increase at ∼ −6 °C, followed by a plateau at lower temperatures. Heating to 30–50 °C causes no measurable changes in the onset temperature or the magnitude of *N*_m_. At 60 and 70 °C, the cumulative freezing spectra remain unchanged, with minor reductions in *N*_m_. At 80 °C, *N*_m_ decreases to ∼10%, and at 90 °C, *N*_m_ decreases by more than 2 orders of magnitude, indicating near-complete loss of ice nucleation activity. Figure 4c compares the cumulative *N*_m_ values and *T*_50_ at the highest measured concentration as a function of temperature. For bacterial INs, *N*_m_ decreases to ∼50% at ∼45 °C and drops further to ∼10% by ∼60 °C, with near-complete loss at 90 °C. In contrast, the fungal INs retain ∼90% activity up to ∼70 °C and reach ∼50% only at ∼75 °C, followed by a sharp decrease to near-complete loss at 90 °C, indicating greater thermal stability of fungal INs.

**Figure 4:**
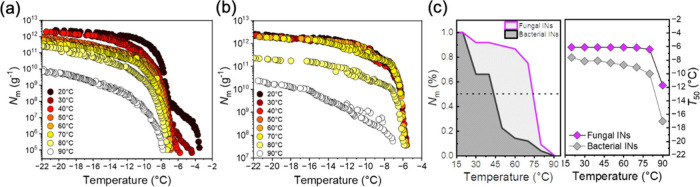
Heat treatment effects on the functionality of INPs. Freezing experiments of aqueous solutions of bacterial INs (a) from *P. syringae* and fungal INs (b) from *E. parvispora* as a function of temperature. (a) Cumulative freezing spectra of a dilution series of bacterial IN in pure water with an initial concentration of 0.1 mg/mL after heating to temperatures ranging from 20 to 90 °C. (b) Cumulative freezing spectra of a dilution series of fungal IN in pure water with an initial concentration of 19 × 10^–5^ mg/mL after heating from 20 to 90 °C. (c) Cumulative number of bacterial and fungal INs and *T*_50_ values at the highest concentration as a function of temperature.

In both cases, *T*_50_ shifts only modestly relative to the large reductions in *N*_m_, highlighting that *T*_50_ values alone underestimate temperature-induced inactivation.^9^ Previous reports on the thermal stability of INPs differ substantially. Davies and co-workers reported apparent heat resistance of bacterial INPs even after heating to temperatures up to 90 °C,^15^ whereas Walsh et al. reported a significant loss of active INs after heating to 95 °C,^28^ and Lukas et al. reported complete inactivation after heating to 120 °C along with a melting transition starting at ∼55 °C as determined by CD spectroscopy.^20^ Our results are consistent with those of Walsh et al. and Lukas et al.,^28,29^ with an observed *N*_m_ midpoint of ∼45 °C that aligns with the proposed structural transition^29^ and supports the interpretation that bacterial INPs undergo temperature-induced destabilization involving changes of the β-solenoid as well as the larger supramolecular aggregates required for efficient ice nucleation. The differing conclusions regarding bacterial INP’s thermal stability likely arise from the data used to assess IN-activity. The findings of Hansen et al. are based on *T*_50_ values,^15^ which reflect the temperature of the most active remaining IN populations and can therefore suggest apparent heat resistance even as substantial losses in the total number of active INs occur. In contrast, *N*_m_-based analysis resolves reductions across the full IN population and is more sensitive to progressive thermal inactivation.

Stabilization of β-solenoid termini has been a persistent challenge in developing stable solenoid folds, as uncapped ends tend to fray or misfold.^2–5^ By directly comparing fungal and bacterial INPs that share a conserved β-solenoid repeat architecture but differ in terminal motifs, we identify disulfide-based terminal capping as a covalent stabilization mechanism that defines β-solenoid stability and function under environmental stress, a solution to the persistent problem of solenoid terminal fraying that has not previously been described.^3,5,6^ In fungal INPs, the disulfide bonds constrain both terminal coils, preserving the continuous surface required for efficient ice nucleation. Reduction of these bonds destabilizes the fold and reduces ice nucleation activity, establishing terminal capping as a key functional determinant of β-solenoid integrity. This differential stabilization of β-solenoids by terminal capping motifs under stress is conceptually consistent with previous observations that ice-binding proteins can facilitate or depress ice nucleation activity depending on environmental conditions.^30^ Prior structural surveys of β-solenoid and β-helix caps have identified loops, hydrophobic contacts, and charged residues as the dominant capping strategies in natural proteins,^1,3^ while engineered solenoids have relied on appended helices, sequence-level redesign of terminal repeats.^5,6^

Disulfide-mediated terminal capping has not been described in this context, but the fungal INP architecture demonstrates that covalent constraints at solenoid termini are not only sufficient for stability but confer environmental robustness unavailable to noncovalent caps. The stability differences between fungal and bacterial INPs are consistent with differences in terminal chemistry, while additional factors such as membrane anchoring, further contribute to bacterial INP instabilities.^18,25,31,32^ Bacterial INPs rely on electrostatic interactions within a charged C-terminal motif together with membrane-association, both of which are sensitive to pH and temperature.^18,25,26^ Fungal INPs employ disulfide bonds at the solenoid termini. These covalent linkages enable retention of ice nucleation activity at pH 12, and a thermal midpoint ∼30 °C higher than that of the bacterial INPs. A recent study of fungal INPs produced by *Podila clonocystis* showed that removal of the N-terminal domain had no significant effect on freezing activity,^28^ suggesting that the α-helical terminus does not play a role in solenoid stability.

These findings provide a molecular context for the greater environmental persistence of fungal INs reported in nature and suggest that covalent terminal constraints may improve the robustness of β-solenoid proteins for applications like cloud seeding or biomolecular templating.^33^ Our results emphasize the importance of reporting cumulative freezing spectra when assessing IN stability. *T*_50_ depends on the probability that a droplet contains at least one active IN and depends on both the underlying IN population and sample concentration. As such, it does not quantify the number of active INs and can therefore not reliably capture partial reductions in IN numbers. Reliance on single-concentration measurements can contribute to inconsistencies in the literature,^15,28^ particularly in studies of highly potent INPs and mutation analyses where reductions in active INs are not accurately reflected by shifts in *T*_50._ ^15,16,28^ More broadly, terminal aggregation or fraying of exposed β-solenoid edges is the primary failure mode in de novo β-solenoid design. The fungal INP structure suggests that adding cysteine pairs at solenoid termini that can form disulfide caps is a promising strategy warranting further testing. While context-specific optimization may be required, this approach may complement or replace the structural caps currently used, enabling β-solenoid scaffolds with both predictable stability and programmable redox-responsive disassembly.

## Supplementary Material



## Notes

Experimental Methods, Figure S1, dilution-resolved effects of alkaline pH on fungal and bacterial INs; Figure S2, T_50_ values after alkaline pH treatments for fungal and bacterial INs; Figure S3, charge state and isoelectric points of fungal and bacterial INs; Figure S4, electrostatic surface map of fungal and bacterial INPs (PDF)
