# Supplementary material for: Design Principles for β‑Solenoid Stability via Covalent and Electrostatic Capping Motifs
Source: J Phys Chem Lett. 2026 May 29;17(24):6730–5. doi: 10.1021/acs.jpclett.6c01287 (PMC13288671; doi:10.1021/acs.jpclett.6c01287)
Supplement: Supplementary file 1 [file jz6c01287_si_001.pdf]

Supporting Information

Design Principles for β-Solenoid Stability via Covalent and Electrostatic Capping Motifs

Authors: R. J. Eufemio[1#], G. Renzer[2#], J. Lehmann[2], S. Gerlach[2], K. Shaw[1], U. Pöschl[3], J. Fröhlich-Nowoisky[3], V. Molinero[4], M. Bonn[2], and K. Meister[1,2*]

Affiliations:

[1]Department of Chemistry and Biochemistry, Boise State University, Boise, ID, 83725

[2]Max Planck Institute for Polymer Research, 55128 Mainz, Germany

[3]Max Planck Institute for Chemistry, 55128 Mainz, Germany

[4]Department of Chemistry, The University of Utah, Salt Lake City, UT 84112-0850

[*]corresponding author: meisterk@mpip-mainz.mpg.de

#equally contributed

Experimental Methods

*Fungal Culture and Sample Preparation***.** 50 plates of the ice nucleation-active fungal species *E. parvispora* were grown on malt yeast extract agar plates (VWR International GmbH). Growth occurred at room temperature for one week and then at 6 °C for about four weeks. Pure water was obtained from Millipore Milli-Q® Integral 3 water purification system (Merck Chemicals GmbH), autoclaved at 121 °C for 15 min, and filtered through a 0.1 µm bottle top filtration unit (VWR International GmbH). For the droplet freezing experiments, mycelial washes of fungal mycelium were prepared as described previously with the following modifications[1,2]. The fungal mycelium of five agar plates was collected in a sterile 50 mL tube, and the weight of the mycelium was determined gravimetrically. Aliquots of 50 mL of pure water were added to the mycelium. The samples were vortexed three times at 2700 rpm for 1 min. The mycelial washes for all experiments were filtered through a 0.1 µm bottle-top filtration unit (VWR International GmbH), and the resulting washes contained ice nucleators from spores and mycelial surfaces. *P. syringae* CiT7 were grown on King B agar for 3 d at 21 °C before assaying, and the concentration of ice nucleators was based on the mass of the bacterial cells. For both the fungal and bacterial samples, the ice nucleator concentration (N_m) was determined by TINA measurements.

TINA Experiments. Ice nucleation experiments were performed using the high-throughput TINA[3]. In a typical experiment, the investigated IN sample was serially diluted 10-fold by a liquid handling station (epMotion ep5073, Eppendorf). Then, 96 droplets (3 µL) per dilution were placed on two 384-well plates and tested with a continuous cooling rate of 1°C/min from 0 °C to −30 °C with a temperature uncertainty of ±0.2 °C. The droplet freezing was determined by two infrared cameras (Seek Therman Compact XR, Seek Thermal Inc.). For each experiment, the obtained fraction of frozen droplets (f_{ice}) and the counting error were used to calculate the cumulative number of IN (N_m) with the associated error using Vali's formula and Gaussian error propagation[4]. All experiments were performed at least three times. Background freezing of pure water in our system occurred at ~−23 °C. We find that independent samples from individual fungal cultures show similar results with minor variations, consistent with previous studies[1,5]. To test the effects of a reducing agent, 100 mM DTT (pH 8) was added to aqueous solutions of *P. syringae* (0.1 mg/mL) and *E. parvispora* (19×10^{-5} mg/mL) and incubated at 37 °C for 20 h. For temperature experiments, the aqueous samples were subjected to temperatures ranging from 20 to 90 °C for 10 min each. For the pH experiments, the pH of the IN samples was adjusted to values ranging from pH 6.5 to 12.6 by adding NaOH. To verify

the reliability of the results obtained under strongly based conditions, additional measurements were performed in buffered solutions at pH 12.0±0.01 (Fisher Scientific, Cat. No. 42425) and 12.5±0.05 (Mettler Toledo, Cat. No. 30464127). These experiments yielded results consistent with those obtained in unbuffered samples.

Prediction of the Protein Structures with AlphaFold3. AlphaFold3 was used for structure predictions [6]. The best-ranked (i.e., with the overall highest predicted local distance difference test (pLDDT) scores) AlphaFold monomer models predict INP as a β-helix with a slight twist. All models were constructed using the same settings (version = 2, modelSeeds = 123).

Circular Dichroism Spectroscopy. The *En*INP sample (lyophilized mycelial wash reconstituted to 3.5 mg/mL in water) was incubated in 20 mM TCEP (pH 6.5) for 15 h at 37 °C and diluted to 0.35 mg/mL prior to CD measurements. CD spectra of the sample were recorded at a 1 nm intervals from 250 – 190 nm using a Jasco J-1500 Spectropolarimeter (JASCO Inc. MD, USA) equipped with a temperature control unit interfaced to a computer. Measurements were performed at 20 °C in a rectangular cell with the optical path of 0.1 cm. Each scan was performed with a scanning speed of 100 nm/min, a data pitch of 0.1 nm, and an accumulation of three. 20 mM TCEP in water (adjusted to pH 6.5) served as the blank and the untreated control was 0.35 mg/mL *En*INP in water (adjusted to pH 6.5). All spectra were background subtracted and the BeStSel webserver was used for secondary structure determination and fold recognition[7].

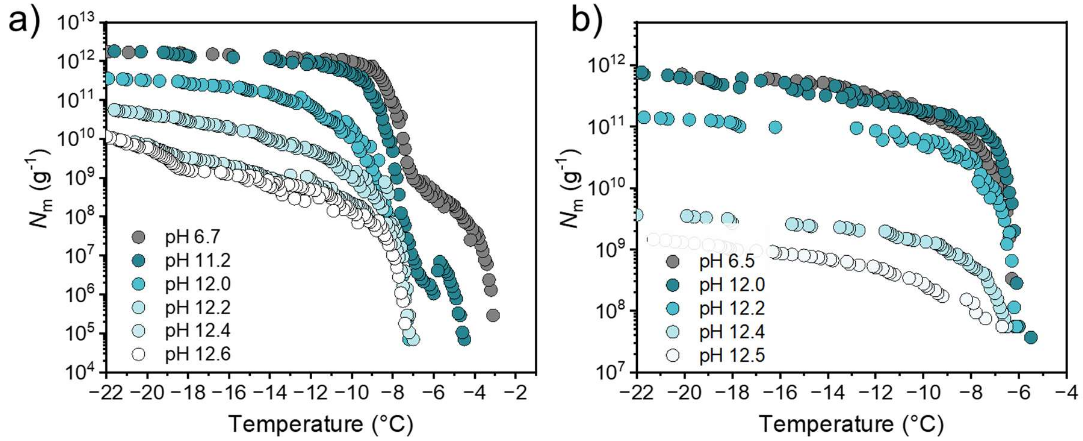

Figure S1. Dilution-resolved effects of alkalinity on the concentration and functionality of INs from bacteria and fungi. Freezing experiments of aqueous solutions of (a) bacterial INs from *P. syringae* and (b) fungal INs from *E. parvispora* at pH ~6.5, 11.2, and 12 – 12.6. Shown are the cumulative number of INs (N_m) per unit mass of sample versus temperature.

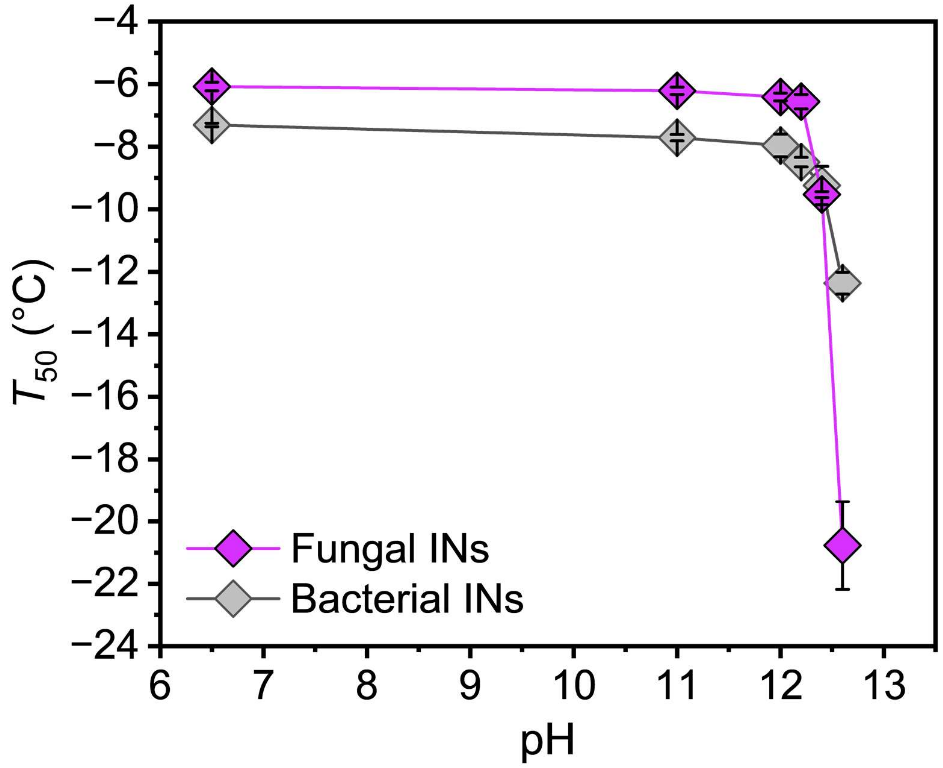

Figure S2. Freezing experiments of fungal INs from *E. parvispora* and bacterial INs from *P. syringae* at pH 6.5 to 12.6. Shown are the T_{50} values of the highest concentration of fungal INs and the concentration of bacterial INs resembling the N_m increase at -7.5°C versus temperature. Error bars represent the standard deviation between individual measurements.

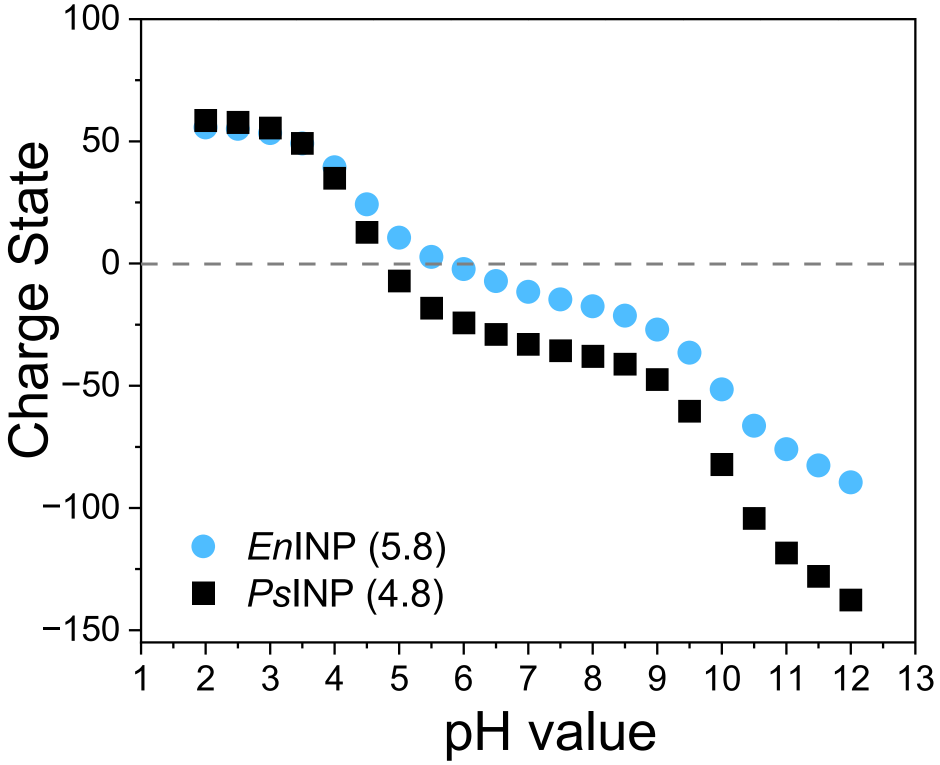

Figure S3. Calculated net charge of the bacterial INP from *P. syringae* (black squares) and of the fungal INPs (blue circles) from *E. parvispora*.

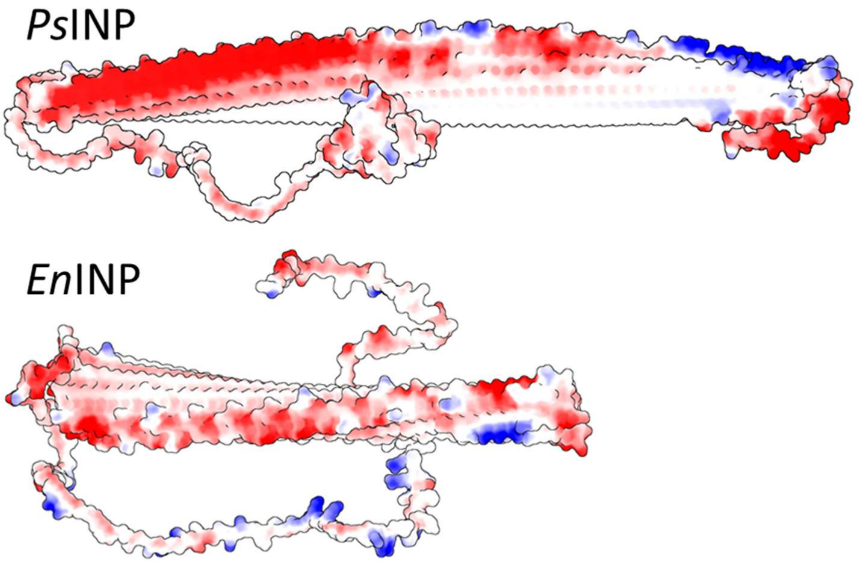

Figure S4. Electrostatic surface maps from the bacterial INP from *P. syringae* and the fungal INP from *E. parvispora*. Positively charged residues (Lysine, Arginine, and Histidine) are shown in shades of blue, while negatively charged residues (Aspartic Acid and Glutamic Acid) are shown in red, respectively. In *Ps*INP, positively charged residues cluster near the C-terminus, while negatively charged residues are found near the N-terminus along one side of the solenoid. In contrast, fungal INPs exhibit a more alternating charge arrangement along the solenoid, with a notable positively charged patch at the N-terminus.

References

(1) Schwidetzky, R.; de Almeida Ribeiro, I.; Bothen, N.; Backes, A. T.; DeVries, A. L.; Bonn, M.; Fröhlich-Nowoisky, J.; Molinero, V.; Meister, K. Functional aggregation of cell-free proteins enables fungal ice nucleation. *Proceedings of the National Academy of Sciences* **2023**, *120* (46), e2303243120. DOI: doi:10.1073/pnas.2303243120.

(2) Kunert, A. T.; Pöhlker, M. L.; Tang, K.; Krevert, C. S.; Wieder, C.; Speth, K. R.; Hanson, L. E.; Morris, C. E.; Schmale Iii, D. G.; Pöschl, U.; et al. Macromolecular fungal ice nuclei in Fusarium: effects of physical and chemical processing. *Biogeosciences* **2019**, *16* (23), 4647-4659.

(3) Kunert, A. T.; Lamneck, M.; Helleis, F.; Pöschl, U.; Pohlker, M. L.; Fröhlich-Nowoisky, J. Twin-Plate Ice Nucleation Assay (Tina) with Infrared Detection for High-Throughput Droplet Freezing Experiments with Biological Ice Nuclei in Laboratory and Field Samples. *Atmos. Meas. Technol.* **2018**, *11* (11), 6327.

(4) Vali, G. Quantitative Evaluation of Experimental Results an the Heterogeneous Freezing Nucleation of Supercooled Liquids. *Int. J. Atmos. Sci.* **1971**, *28* (3), 402.

(5) Fröhlich-Nowoisky, J.; Hill, T. C. J.; Pummer, B. G.; Yordanova, P.; Franc, G. D.; Pöschl, U. Ice nucleation activity in the widespread soil fungus Mortierella alpina. *Biogeosciences* **2015**, *12* (4), 1057-1071. DOI: 10.5194/bg-12-1057-2015.

(6) Abramson, J.; Adler, J.; Dunger, J.; Evans, R.; Green, T.; Pritzel, A.; Ronneberger, O.; Willmore, L.; Ballard, A. J.; Bambrick, J.; et al. Accurate structure prediction of biomolecular interactions with AlphaFold 3. *Nature* **2024**, *630* (8016), 493-500. DOI: 10.1038/s41586-024-07487-w.

(7) Micsonai, A.; Moussong, É.; Wien, F.; Boros, E.; Vadászi, H.; Murvai, N.; Lee, Y.-H.; Molnár, T.; Réfrégiers, M.; Goto, Y.; et al. BeStSel: webserver for secondary structure and fold prediction for protein CD spectroscopy. *Nucleic Acids Research* **2022**, *50* (W1), W90-W98. (acccessed 5/15/2023).
